# Editorial

**DOI:** 10.1186/2046-2530-3-8

**Published:** 2014-08-18

**Authors:** Lotte B Pedersen

**Affiliations:** 1 Department of Biology, University of Copenhagen, Copenhagen, Denmark

## 

Intraflagellar transport (IFT) is a bidirectional microtubule-based trafficking system required for the assembly and maintenance of almost all eukaryotic cilia and flagella^a^. IFT was discovered in 1993 by Keith Kozminski and colleagues in Joel Rosenbaum’s laboratory at Yale University; by employing enhanced digital interference contrast microscopy of immobilized flagella of the green alga *Chlamydomonas*, Kozminski and colleagues observed IFT for the first time as a rapid movement of granule-like particles beneath the flagellar membrane and along the length of the axoneme, from its base to the tip and back [1].

This first observation of IFT was a landmark discovery that has been instrumental for understanding the molecular mechanisms of cilium assembly, and which led to tremendous progress in our understanding of cilia biology and function. Perhaps one of the most significant advancements in cilia biology during the past two decades is the realization that cilia not only play important roles in cell motility or sensory perception in specialized cell types like photoreceptors and olfactory neurons, but are involved in regulating various signaling pathways and developmental processes throughout most of our body. Consequently, it is now clear that a growing number of pleiotropic diseases called ciliopathies are caused by mutations in genes that affect cilia assembly or function.

Since cilia are compartmentalized organelles that lack the machinery for protein synthesis, cilia assembly requires transport of ciliary precursors from their site of synthesis in the cell body to the ciliary base, selective entry of precursors to the ciliary compartment, followed by their delivery to the appropriate sub-ciliary location by IFT.

This series in *Cilia* celebrates the discovery of IFT with a collection of original research articles and reviews focused on different aspects of IFT and cilia assembly mechanisms.

The IFT system consists of large IFT particles comprising about 22 different IFT particle polypeptides organized in two different complexes, IFT-A and IFT-B, as well as kinesin-2 and cytoplasmic dynein 2 motors that drive anterograde (base to tip) and retrograde (tip to base) IFT, respectively. In their review ‘Intraflagellar transport complex structure and cargo interactions’, Bhogaraju and colleagues [2] provide an update on IFT complex structure and architecture and discuss how the IFT complex polypeptides may interact with motors and cargo to regulate cilium assembly and function.

Much of our current knowledge about IFT and mechanisms of cilium assembly stems from studies carried out in model organisms such as *Chlamydomonas* and *Trypanosoma*. In their review entitled ‘Getting to the heart of intraflagellar transport using *Trypanosoma* and *Chlamydomonas* models: the strength is in their differences’, Morga and Bastin [3] examine and compare data available from these organisms, highlight pertinent questions about IFT, and discuss specific biological and experimental advantages of these two powerful models.

Two original research articles illustrate the power of *Chlamydomonas* for studying cilium assembly. First, in their article ‘New mutations in flagellar motors identified by whole genome sequencing in *Chlamydomonas*’, Lin and colleagues [4] identify by whole genome sequencing in *Chlamydomonas* the causative mutations for two temperature-sensitive flagellar assembly mutants and use these mutants to gain new insight into the molecular mechanisms of IFT. Second, in their article ‘Flagellar central pair assembly in *Chlamydomonas reinhardtii*’, Lechtreck and colleagues [5] use specific *Chlamydomonas* mutants to study the mechanisms of axonemal central pair microtubule assembly by electron microscopy and wide-field and super-resolution immunofluorescence microscopy.

Vertebrate model systems have also proved very useful in cilia studies, and in their original research article entitled ‘The Small GTPase Rsg1 is important for the cytoplasmic localization and axonemal dynamics of intraflagellar transport proteins’ Brooks and Wallingford [6] use the *Xenopus* model to explore the role of the small GTPase Rsg1 in ciliogenesis. They find that Rsg1 plays an important role in regulating IFT dynamics and IFT polypeptide localization in multiciliated cells, as well as in apical localization of basal bodies that anchor cilia.

Transport of ciliary precursors from their site of synthesis in the cell body to the cilium base is complex and involves multiple regulatory factors, adaptors and coat proteins that control vesicular trafficking, docking, and fusion. In their original research article entitled ‘Identification of conserved, centrosome-targeting ASH domains in TRAPPII complex subunits and TRAPPC8’, Schou and colleagues [7] use bioinformatics to identify conserved ASPM, SPD-2, Hydin (ASH) domains in specific transport protein particle (TRAPP) complex subunits, several of which were previously implicated in ciliogenesis and vesicle trafficking to the ciliary base, and further demonstrate using cultured mammalian cells that the ASH domain confers targeting to the centrosome, and that the TRAPP subunit TRAPPC8 localizes to the basal body and is involved in ciliogenesis.

Following transport of ciliary precursors from their site of synthesis in the cell body to the ciliary base, precursor proteins need to cross the ciliary transition zone to enter the ciliary compartment. Recent research has indicated that the mechanisms by which cytosolic proteins are imported to the ciliary compartment are similar to the mechanisms of nuclear import, as discussed by Kee and Verhey in their review entitled ‘Molecular connections between nuclear and ciliary import processes’ [8].

The ciliary axoneme is a direct extension of the basal body, which in monociliated cells, for example, a cell with a non-motile primary cilium, is derived from the mother centriole of the centrosome. During mitosis, centrosomes function as mitotic spindle poles, which means that ciliogenesis needs to be tightly coordinated with the cell cycle. Indeed, in most cells cilia are resorbed prior to mitosis and re-form when cells enter G1/G0. To prevent untimely assembly of cilia, cells have devised mechanisms to precisely control when to initiate ciliogenesis. One critical molecule involved in this process is the centriolar protein CP110, and in their review entitled ‘CP110 and its network of partners coordinately regulate cilia assembly’ Tsang and Dynlacht [9] discuss recent advances in our understanding of the regulatory mechanisms underlying cilia assembly, with focus on CP110 and its interaction partners.

We hope that this special article series on Cilia assembly mechanisms will be of interest to many readers and stimulate discussions and further research within the fascinating field of cilia biology - a field that has already attracted increased attention in recent years following the seminal discovery of IFT by Kozminski and colleagues two decades ago.

## Endnotes

^a^Cilia and flagella are equivalent organelles.

## References

[B1] Kozminski KG Johnson KA Forscher P Rosenbaum JL A motility in the eukaryotic flagellum unrelated to flagellar beating Proc Natl Acad Sci U S A 1993 90 5519 5523 10.1073/pnas.90.12.5519 8516294 PMC46752

[B2] Bhogaraju S Engel BD Lorentzen E Intraflagellar transport complex structure and cargo interactions Cilia 2013 2 10 10.1186/2046-2530-2-10 23945166 PMC3751104

[B3] Morga B Bastin P Getting to the heart of intraflagellar transport using Trypanosoma and Chlamydomonas models: the strength is in their differences Cilia 2013 2 16 10.1186/2046-2530-2-16 24289478 PMC4015504

[B4] Lin H Nauman NP Albee AJ Hsu S Dutcher SK New mutations in flagellar motors identified by whole genome sequencing in Chlamydomonas Cilia 2013 2 14 10.1186/2046-2530-2-14 24229452 PMC4132587

[B5] Lechtreck KF Gould TJ Witman GB Flagellar central pair assembly in Chlamydomonas reinhardtii Cilia 2013 2 15 10.1186/2046-2530-2-15 24283352 PMC3895805

[B6] Brooks ER Wallingford JB The Small GTPase Rsg1 is important for the cytoplasmic localization and axonemal dynamics of intraflagellar transport proteins Cilia 2013 2 13 10.1186/2046-2530-2-13 24192041 PMC3850895

[B7] Schou KB Morthorst SK Christensen ST Pedersen H Identification of conserved, centrosome-targeting ASH domains in TRAPPII complex subunits and TRAPPC8 Cilia 2014 3 6 10.1186/2046-2530-3-6 25018876 PMC4094338

[B8] Kee HL Verhey KJ Molecular connections between nuclear and ciliary import processes Cilia 2013 2 11 10.1186/2046-2530-2-11 23985042 PMC3765448

[B9] Tsang WY Dynlacht BD CP110 and its network of partners coordinately regulate cilia assembly Cilia 2013 2 9 24053599 10.1186/2046-2530-2-9 PMC3744162

